# How we read FCH-PET/CT for prostate cancer

**DOI:** 10.1186/s40644-016-0101-5

**Published:** 2016-12-06

**Authors:** Jean-Mathieu Beauregard, Alexis Beaulieu

**Affiliations:** 1 Department of Medical Imaging, CHU de Québec – Université Laval, 11 côte du Palais, Quebec City (QC), G1R 2J6 Canada; 2 Department of Radiology and Nuclear Medicine, Université Laval, Quebec City, Canada; 3 Oncology Branch, CHU de Québec – Université Laval Research Center, Quebec City, Canada

**Keywords:** ^18^F-fluorocholine, FCH, PET/CT, Prostate cancer, Interpretation

## Abstract

Over the last decade, ^18^F-fluorocholine positron emission tomography/computed tomography (FCH-PET/CT) has gained in popularity for the staging and restaging of patients with prostate cancer (PCa). However, despite abundant literature on the topic, there is a lack of publications on how to actually interpret FCH-PET/CT in a clinical setting. Here we propose a practical, TNM-oriented approach to read FCH-PET/CT, with notes on procedure technique, image display, review sequence and report structure. The purpose of this article is to provide guidance to radiologists, nuclear medicine physicians and residents who are new to FCH-PET/CT, as well as to propose an alternate approach to more experienced physicians.

## Background

Positron emission tomography/computed tomography (PET/CT) with radiolabeled choline, a marker of cell membrane synthesis, has proven to be a useful imaging technique in the management of prostate cancer (PCa). ^18^F-fluorocholine (FCH) is more convenient than ^11^C-choline, the tracer of reference, as it can be delivered to centers devoid of an on-site cyclotron, owing to the longer half-life of ^18^F. ^11^C-choline is now FDA-approved for restaging of PCa in the setting of biochemical relapse after radical prostatectomy [1], and this indication is also the most established one for FCH-PET/CT to date [2–4]. FCH-PET/CT is also useful for initial stating of patients with high-risk PCa [2, 4]. FCH usually refers to ^18^F-fluoromethylcholine, but ^18^F-fluoroethylcholine has also been employed. Both tracers appear to have a comparable biosdistribution [5], although ^18^F-fluoromethylcholine has been used and studied more extensively [6]. The reading approach presented here applies to both tracers.

## Technique

Contrary to ^18^F-fluorodeoxyglucose (FDG)-PET/CT, fasting prior to FCH-PET/CT is not required, as FCH biodistribution and tumor uptake are not affected by glycemia or insulinemia. Androgen deprivation therapy (ADT) does not need to be stopped before FCH-PET/CT performed in the setting of biochemical recurrence under ADT, as castration-resistant PCa lesions are proliferating despite ADT [7].

Tumor uptake and blood clearance of FCH are rapid. Because of this a prolonged uptake period, such as 60 min when doing an FDG-PET/CT, is not mandatory with FCH. At our center, FCH is rather injected on the scanner table while simultaneously starting a 10-min dynamic acquisition over the pelvis. This allows visualization of early pathological uptake in the prostate or the prostatic bed, as well as in the regional nodal stations, before the urine activity enters the distal ureters and bladder, thus avoiding the need for a delayed or post-diuretic acquisition, or bladder catheterism. The dynamic pelvic acquisition can be reconstructed in fixed (e.g. 5 × 2 min) or progressively longer frames (e.g. 4 × 30 s, 4 × 1 min and 2 × 2 min).

The dynamic acquisition is immediately followed by the whole-body (WB) acquisition from skull vertex to proximal thighs, with arms preferably upward. It is rarely necessary to include the distal upper or lower limbs, unless there are known or suspected metastases beyond proximal part thereof. Contrary to FDG-PET/CT however, skull and brain are systematically included in FCH-PET/CT acquisition because (1) skull is a frequent site of axial bone metastases in PCa, and (2) brain is much easier to assess than with FDG-PET/CT owing to the lack of physiological FCH uptake in the normal brain. Oral contrast can optionally be administered to better discriminate intestinal loops and confirm physiological uptake or pancreatic excretion of FCH along the gastrointestinal tract.

## Image display

### Template

**Fig. 1:**
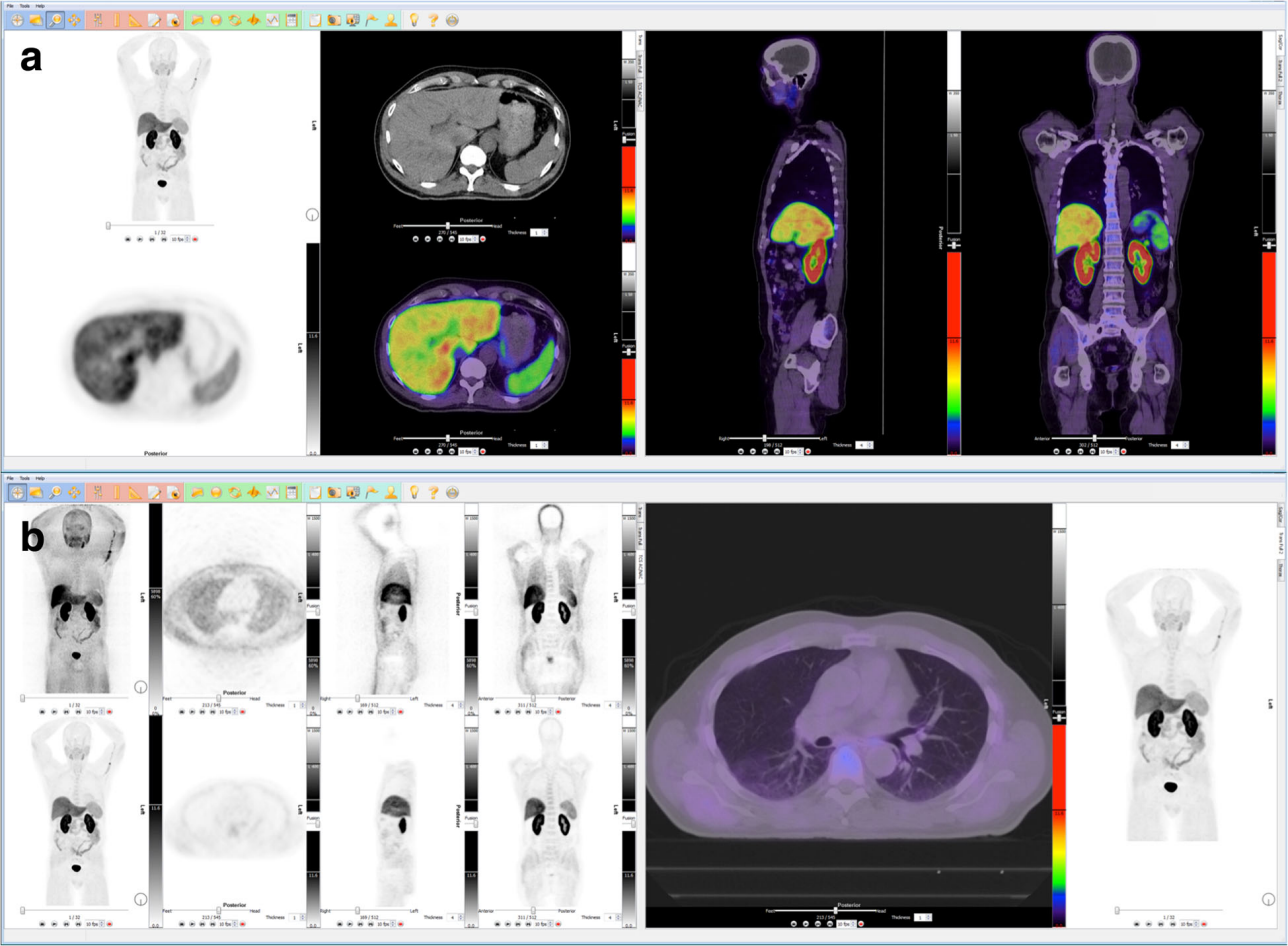
Dual-screen whole-body FCH-PET/CT image display template (Hybrid Viewer, Hermes Medical Solutions, Stockholm, Sweden). The primary tab (**a**) includes AC PET MIP plus CT, PET and fused PET/CT transaxial views on the left screen, and fused sagittal and coronal views on the right screen. The secondary tab (**b**) includes MIP and orthogonal views of NAC and AC PET on the left, and a large transaxial PET/CT view on the right. A rainbow color LUT (Siemens ECAT Rainbow) is used for the fused images, which are displayed with a linear, 50–50% blend. AC PET imaged intensity is normalized by adjusting the upper limit of the color scale so that the liver is nearly, but not, saturated (mostly yellow to orange). The same SUV upper threshold is used to adjust the inverse gray scale intensity of the PET-only images, as well as the dynamic PET images. This is a case of a negative FCH-PET/CT in a patient with biochemical relapse after radical prostatectomy, where physiological FCH distribution is seen

At our center, WB FCH-PET/CT images are displayed using the same viewing template and settings as for FDG-PET/CT (Fig. 1). A viewing template that is not too crowded allows maximizing the use of screen estate to display the images with a decent zoom. Some nuclear medicine software viewers offer a tab organization. In our PET/CT template, the primary tab contains an attenuation-corrected (AC) PET rotating maximum intensity projection (MIP) view, 3-plane orthogonal fused PET/CT views (transaxial, coronal, sagittal) and a CT-only transaxial view (Fig. 1a). The non attenuation-corrected (NAC) PET images are conveniently placed in a secondary tab, alongside AC PET (Fig. 1b). The dynamic images (at least AC PET MIP and orthogonal fusion views) are displayed in an additional tab or a separate template, depending on software capabilities.

### PET color maps and fusion blend

The PET-only images, including MIP, are displayed with an inverse linear gray look-up table (LUT) color map. When fused with CT, a rainbow LUT (e.g. “ECAT Rainbow” found on Siemens’ systems; Fig. 1) is preferred, as it provides more contrast over a wider dynamic range than LUTs incorporating fewer colors (such as red and yellow “Hot Metal” LUT). A rainbow LUT starting with black naturally attenuates the unwanted low-level background noise. The upper end of the LUT should preferably not be white, but rather of a bright color such as red, in order to minimize the interference with the underlying black and white CT signal. Furthermore, a desired feature of the chosen rainbow LUT is a logarithmic progression, which naturally allows distinguishing uptake level differences that are clinically meaningful, both in the lower and the higher ends of the uptake intensity spectrum. A rainbow LUT with these characteristics facilitates performing most of the orthogonal slices review in fusion mode. A linear blending mode with an alpha blend of 50% should be used in fusion mode (Fig. 1), so that both CT and PET images are equally represented and neither modality is ever masked by the other, particularly in areas of high uptake or density.

### PET images normalization and visual uptake assessment

Unlike FDG, FCH has a relatively high physiological uptake in the liver. Because of this, FCH images are normalized by making the liver appear yellow to orange, on average, and not saturated (Fig. 1). We find that adjusting the inverse gray scale LUT of PET-only images to the same upper standardized uptake value (SUV) threshold as the color LUT provides an adequate normalization thereof, with the same dynamic range as color PET images. Once the whole-body PET is properly normalized, the dynamic pelvic PET images (which do include the liver) are normalized to the same upper SUV threshold as that of the WB PET images.

As with FDG-PET, normalizing FCH-PET images using a fixed upper SUV threshold in all patients is not advisable because of: (a) the inter-patient variation in biodistribution and kinetics, (b) the weight bias affecting SUVs, (c) potential technical errors such as erroneous entry of weight, FCH dose and injection time, and (d) scanner differences that can also affect SUVs. Rather, the use of physiological uptake landmarks, such as that of the liver and, in the case of FCH, that of exocrine glands, appears a more rational way to normalize images consistently, both across patients and within the same patient. Furthermore, such an approach to normalization can help harmonizing inter-observer readings within and between centers. Uptake intensity is easily qualitatively described by comparing with such physiological landmarks (Table 1), in a similar fashion that we have described for ^18^F-fluorothymidine PET [8].

**Table 1:** Proposed FCH visual uptake scale for PCa lesions

Score	Uptake	Qualitative terms	Large lesion (> ~ 1 cm)	Small lesion^a^ (≤ ~1 cm)
0	Not visible (equal to or lower than background)	No uptake	-	-
1	Higher than background, but lower than that of the bone marrow	Faint uptake	Bluish	-
2	Similar to or higher than that of the bone marrow, but lower than that of the liver and exocrine glands^b^	Mild uptake	Greenish	Bluish
3	Similar to or between that of the liver and exocrine glands	Moderate uptake	Yellowish	Greenish
4	Higher than that of the liver and exocrine glands	Intense uptake	Reddish	Yellowish
5	Comparable to or higher than that of the kidney	Very intense uptake	Red (large area of saturation)	Reddish

^a^For smaller lesions, the uptake score is upgraded (vs. larger lesions) to account for partial volume effect

^b^Pancreas and salivary glands

## Normal FCH biodistribution

Before attempting to interpret FCH-PET/CT, one must be familiar with the normal biodistribution and kinetics of the tracer, which has been covered in details elsewhere [9]. On the dynamic scan, the first-pass vascular bolus is noted in the iliac arteries on the first frame(s) and rapidly fades away. Urine activity typically reaches distal ureters and the bladder at around 4 to 6 min, but sometimes later.

Kidneys have a very intense physiological uptake, usually the highest among healthy organs, and are almost always saturated when PET images are normalized as above. Liver uptake is quite intense, but less than that of the kidneys. The uptake in spleen and major exocrine glands (pancreas, salivary and lachrymal glands) is usually moderate and lower than that of the liver, but sometimes comparable or slightly above the latter. Bone marrow and endocrine glands (pituitary, thyroid, adrenals) generally show faint to mild uptake. Uptake in the gastro-intestinal tract is highly variable in extent and intensity, and is related to the rapid turnover of mucosal cells, in addition to probable excretion of FCH within pancreatic juices (hypothesized from our frequent observation of continuous activity extending from the duodenum along downstream small bowel). Skeletal and cardiac muscles, as well as choroid plexus exhibit faint and diffuse uptake. Marked uptake is frequently seen along the vein proximal to injection site (Fig. 1). The normal prostate exhibits minimal uptake.

## Interpretation

### The TNM approach

A problem-oriented approach to reading FCH-PET/CT (and oncological PET/CT in general) is better achieved by adopting a tumor-node-metastasis (TNM) sequence than using the typical radiological anatomical segmentation (i.e. head and neck, thorax, abdomen and pelvis). Indeed, PET/CT being a systemic imaging primarily used to rule in or out a systemic disease (i.e. the presence or not of metastasis), compartments delimited by anatomic boundaries such as the thoracic inlet or the diaphragm make little sense when describing the extent of PCa disease. Indeed, beginning with description of findings in head and neck area, while PCa actually originates from the pelvis and spreads upward, is counter-intuitive. Furthermore, bone, the most frequent site of distant metastasis in PCa, is an organ present in all radiological segments and is more logically subdivided into axial and appendicular skeleton in the oncological setting. In fact, bone scan is seldom interpreted using conventional cross-sectional anatomical divisions, as bone metastases do not recognize those arbitrary boundaries. The same logic applies to FCH-PET/CT.

Accordingly, in the context of PCa, we begin by describing findings in prostate or prostatic bed, then those in proximal to distal nodal stations relative to the prostate, and finally those at potential sites of distant metastases starting with the most frequent, the skeleton. For each TNM station, a clear and concise description of PCa-relevant positive and negative findings is warranted. This is followed by description of incidental findings, if any, finishing with accessory findings.

### Image review order

Although the dynamic acquisition is performed first, we find it more efficient to begin by reviewing the WB scan. The first series to look at when opening the FCH-PET/CT study is the WB AC-PET MIP, which is the most informative and fastest to assess PET-only representation. In just a few seconds, is it possible in the vast majority of cases to determine the M and even N statuses on the MIP. Even the subtlest foci of uptake can be seen in at least a few projection angles, and can then easily be located onto the orthogonal views with a simple mouse click. When distant metastases are present (M1), the patient is generally inoperable. Having this information in mind from the start will allow the reader to be more concise and clinically relevant when describing the loco-regional findings. Contrary to conventional cross-sectional imaging, PET MIP allows looking at the big picture in a trice, i.e. seeing the forest for the trees. In a patient with numerous metastases, the emphasis will be on that big picture (e.g. overall tumor burden, sites involved, metabolic intensity and heterogeneity, etc.), rather than on minute loco-regional details or exact number of lesions, which would be more important to emphasize in a surgical case.

Following the general survey based on MIP, we proceed with a careful review of PET/CT fused transaxial slices (referring to coronal and sagittal slices as needed). CT-only images are consulted as needed for better locating, delineating or measuring lesions of interest. Throughout reading, CT windowing is alternated between soft tissue, bone, lung and brain windows as appropriate. We find that orthogonal AC PET-only slices are less often consulted because rotating MIP already provides an excellent display of PET alone in a very practical format. However, PET-only slices are sometimes helpful to distinguish between what constitutes a definite focus of mild uptake that may suggest the presence of a small lesion, versus non-specific heterogeneity or statistical noise.

NAC PET images are useful for quality control purposes, and to assess areas or abnormalities prone to attenuation correction artifacts and misregistration. Review of lung fields on NAC PET, where they are naturally overrepresented, can improve the sensitivity to detect or confirm small foci of uptake. We therefore tend to review NAC images at the time of surveying lungs for metastases. After going through the TNM sequence, as described below, there will be a rapid, general survey of CT-only transaxial slices, looking for potential incidental or accessory findings not already identified on PET and fused images.

### T: Local compartment assessment

**Fig. 2:**
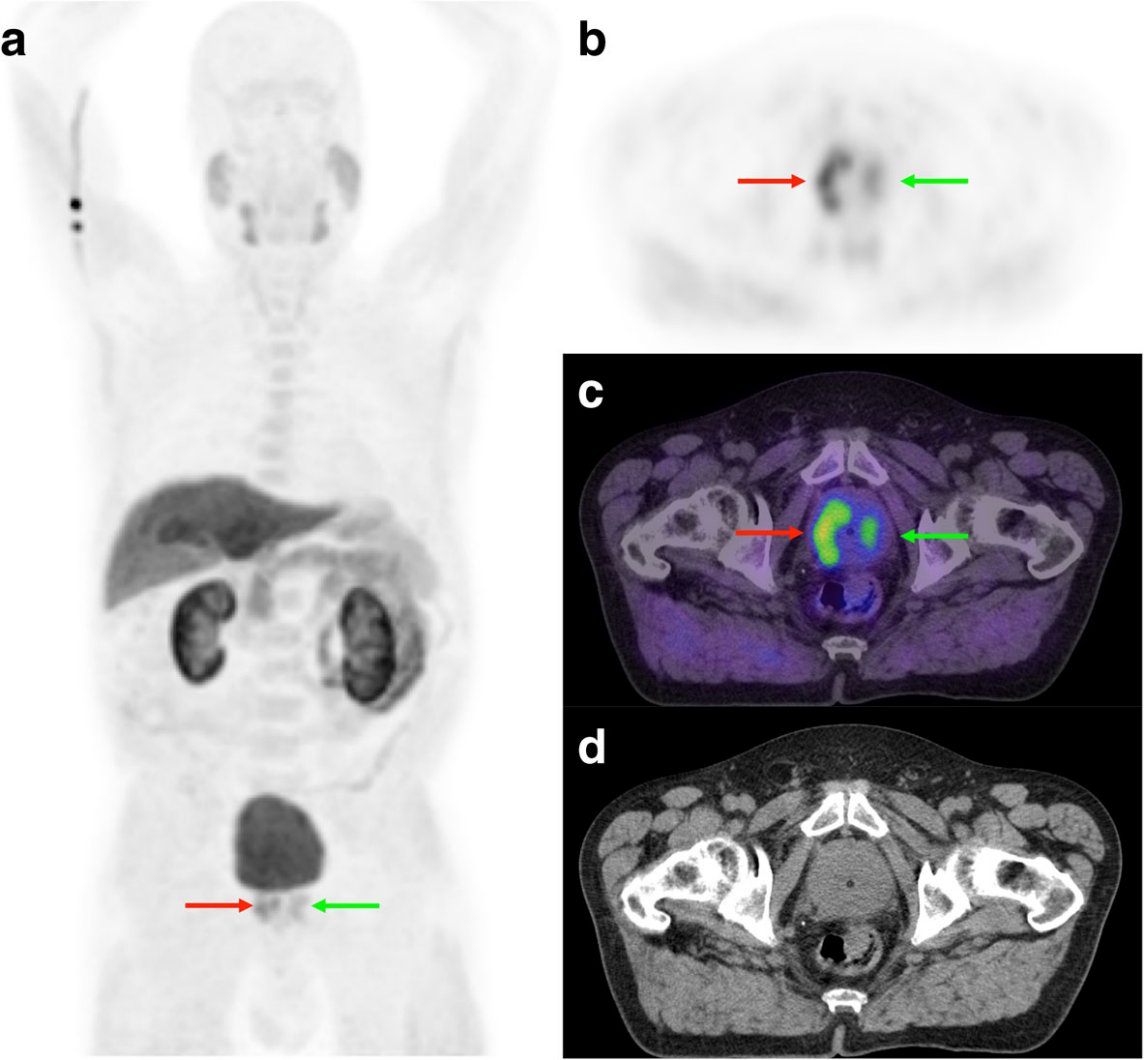
FCH-PET/CT for staging and radiotherapy planning in a 78 y.o. patient with biopsy-proven PCa. Biopsy Gleason sum was 7 and PSA was 13 mg/L. MIP (**a**) and transaxial PET (**b**), fusion (**c**) and CT (**d**) views showed a moderately FCH-avid, multi-focal dominant intra-prostatic lesion in the right prostatic lobe (red arrows), consistent with a primary PCa lesion and the biopsy findings. A second area of milder focal uptake was seen in the left prostatic lobe (green arrows) and raised some suspicion for a contralateral PCa lesion. However, both the initial biopsy and a repeat biopsy were negative for the presence of PCa in that area, and the patient received high dose-rate brachytherapy to the right dominant lesion only, in addition to external radiotherapy

**Fig. 3:**
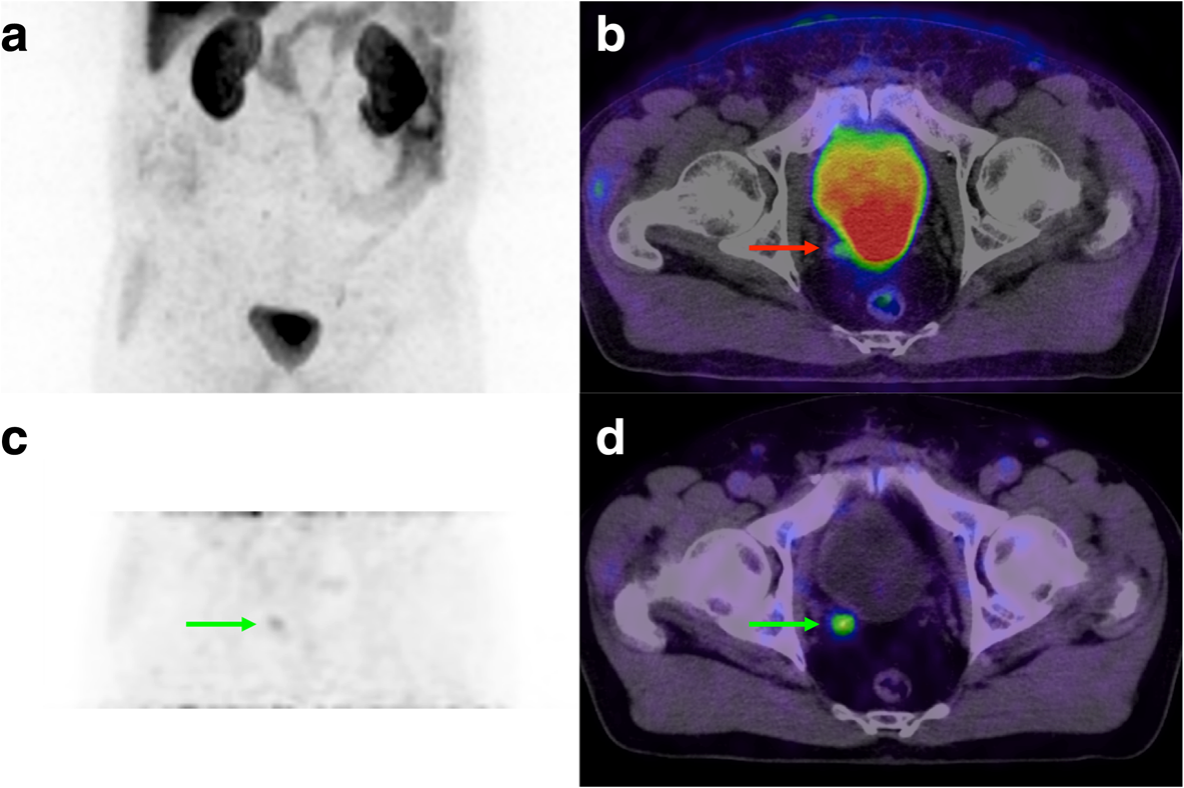
Restaging FCH-PET/CT in a 72 y.o. patient with biochemical relapse following radical prostatectomy. Initially, PCa Gleason sum was 9 and left seminal vesicle was involved. PSA at time of examination was 1.6 mg/L. On whole-body MIP (**a**) and transaxial PET/CT fused slice (**b**), a focus of uptake was not clearly distinguishable from the intense urinary activity in the bladder (red arrow). However, corresponding images from the dynamic PET/CT (**c, d**) clearly showed the early appearance of a focus of moderate FCH uptake in right prostatic bed, before apparition of urinary activity and consistent with a local relapse (green arrows). There was no evidence of metastasis and the patient was treated with salvage radiotherapy

When FCH-PET/CT is performed for primary staging, after PCa has been proven by biopsy, we tend to review the prostate gland with a relatively high sensitivity. Because there is proven malignancy in the prostate, any well-defined focus of uptake raises high suspicion of malignancy, regardless of biopsy results in the concerned sextant(s). Indeed, PCa is frequently multifocal, often bilateral, and biopsy sampling errors are common [10]. However, prostatitis and benign prostate hyperplasia have been reported as benign causes of increased FCH uptake, and consequently correlations between intra-prostatic FCH uptake foci and PCa lesions distribution have yielded mixed results [2, 4]. Nevertheless, finding that a dominant lesion is located in an area that was not reported as involved on core biopsy may trigger the decision to re-biopsy if this may alter patient management (e.g. in the planning of brachytherapy; Fig. 2). In the setting of biochemical relapse following radiotherapy or prostatectomy, a definite focus of uptake in the prostate or prostatic bed, respectively, would be suspicious for local recurrence. Otherwise, normal prostate uptake is usually faint. Any finding in the prostate area or bed on the WB PET must be confronted to the pelvic dynamic series, particularly if a focal uptake abnormality is located centrally in the prostate or at its base, and susceptible to represent urinary activity (Fig. 3). We qualitatively describe the uptake intensity of the suspicious lesions (Table 1), their location and extent (intraprostatic location, seminal vesicle involvement, extraprostatic extension), their approximate size based on PET (unless clearly measurable on CT). We also comment on the signs of potential local complications caused by the primary tumor, if any, such as bladder or rectum involvement or urinary obstruction.

### N: Regional compartment assessment

**Fig. 4:**
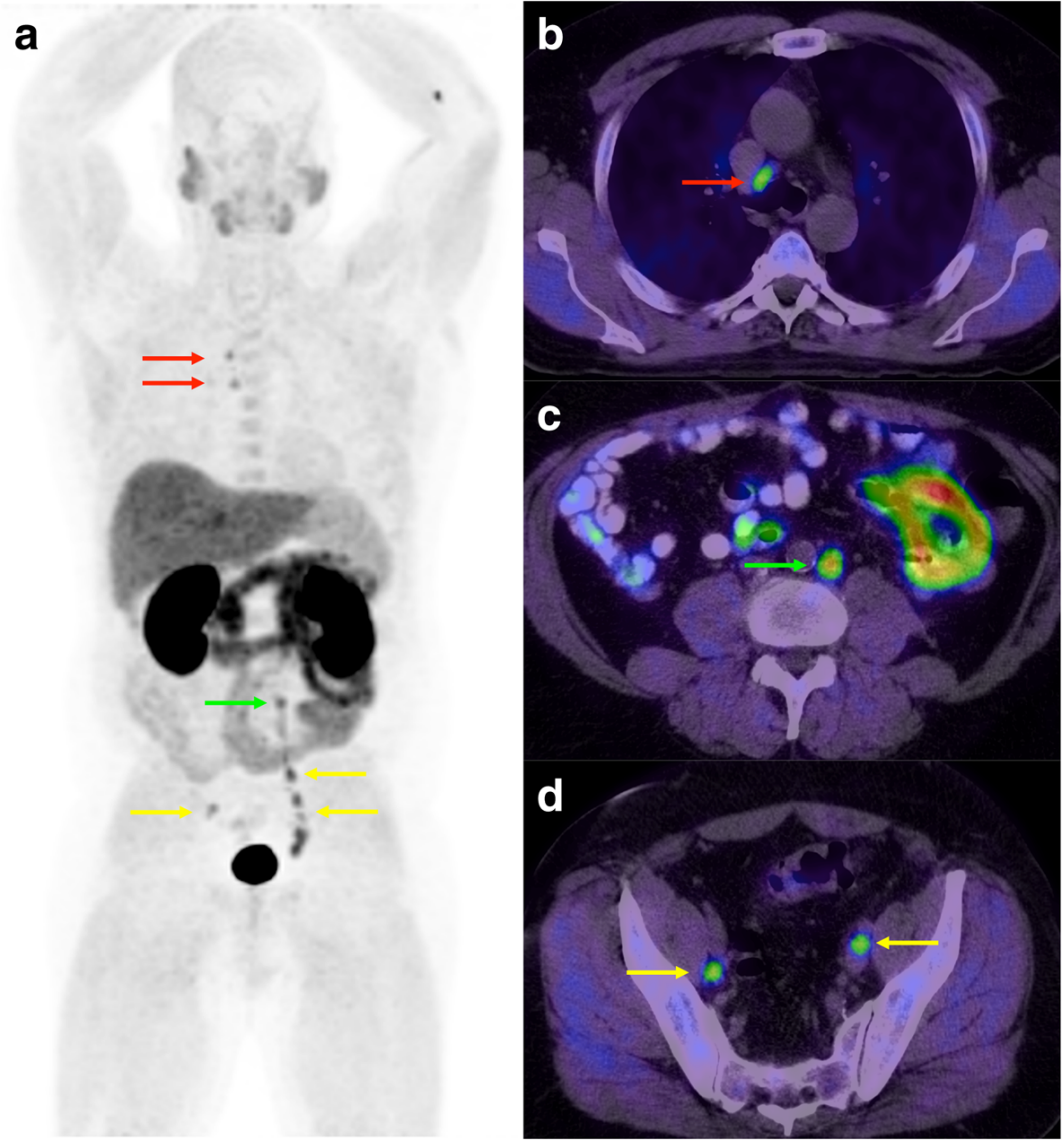
Restaging FCH-PET/CT in a 58 y.o. patient with biochemical relapse following external beam radiotherapy. Gleason sum was 9 on initial biopsy. PSA at time of examination was 29.3 mg/L. Whole-body MIP (**a**) and transaxial fusion series (**b, c, d**) showed intense uptake in metastatic lymph nodes in the right paratracheal (red arrows), left para-aortic (green arrows) and bilateral iliac areas (yellow arrows)

**Fig. 5:**
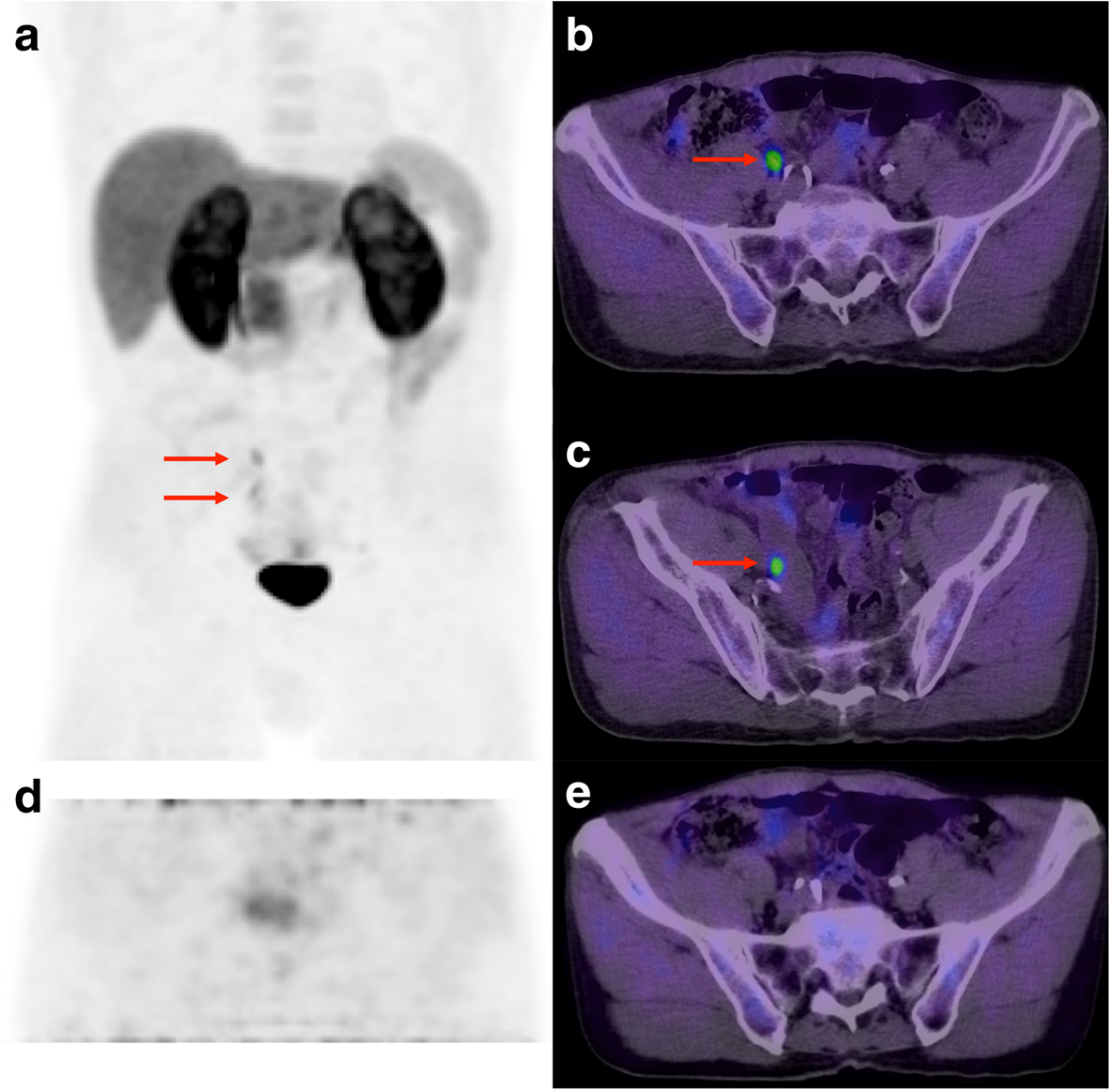
Restaging FCH-PET/CT in a 68 y.o. patient with biochemical relapse following radical prostatectomy. Initially, PCa Gleason sum was 7. PSA was 0.35 mg/L at time of examination. Whole-body MIP (**a**) and transaxial fusion slices (**b, c**) showed two foci of moderate uptake in the right iliac nodal area (red arrows) that were initially perceived as suspicious for nodal relapse. However, dynamic PET MIP (**d**) and fusion series (**e**) showed no early focal activity at either site, which was consistent with urinary activity on the whole-body scan. This study was interpreted as negative and the patient underwent salvage radiotherapy

PCa most commonly spreads to the periprostatic and external iliac nodes (including obturator nodes) before progressing further to non-regional nodes. Dissemination through internal iliac and perirectal stations is also possible and is considered regional spread as well. Most of the time at least one of these nodal stations is involved before PCa spreads upward to non-regional nodes (common iliac, retroperitoneal, mediastinal, supraclavicular) or occasionally downward to inguinal nodes (suggesting lymph flow disturbance because of metastatic involvement) (Fig. 4). In the setting of restaging following radiotherapy or pelvic lymph node dissection, the pattern of nodal disease may differ, as the probability of finding distant nodal metastases in the absence regional relapse is higher. Nodal metastases tend to be ipsilateral to the primary lesion when unilateral. In FCH-PET, benign nodes have the same pattern as that commonly seen in FDG-PET: axillary, inguinal and mediastinal/hilar nodes having a variable degree of uptake and which are most often symmetric in distribution. As such, isolated hyperactive inguinal nodes are almost always benign (inflammatory or so called “reactive”), even more so if bilateral. Dynamic PET can help distinguishing nodal uptake from urinary activity in the ureter, particularly when the latter is focal instead of curvilinear, as often seen at the crossing of the ureter over the iliac vessels (Fig. 5).

**Fig. 6:**
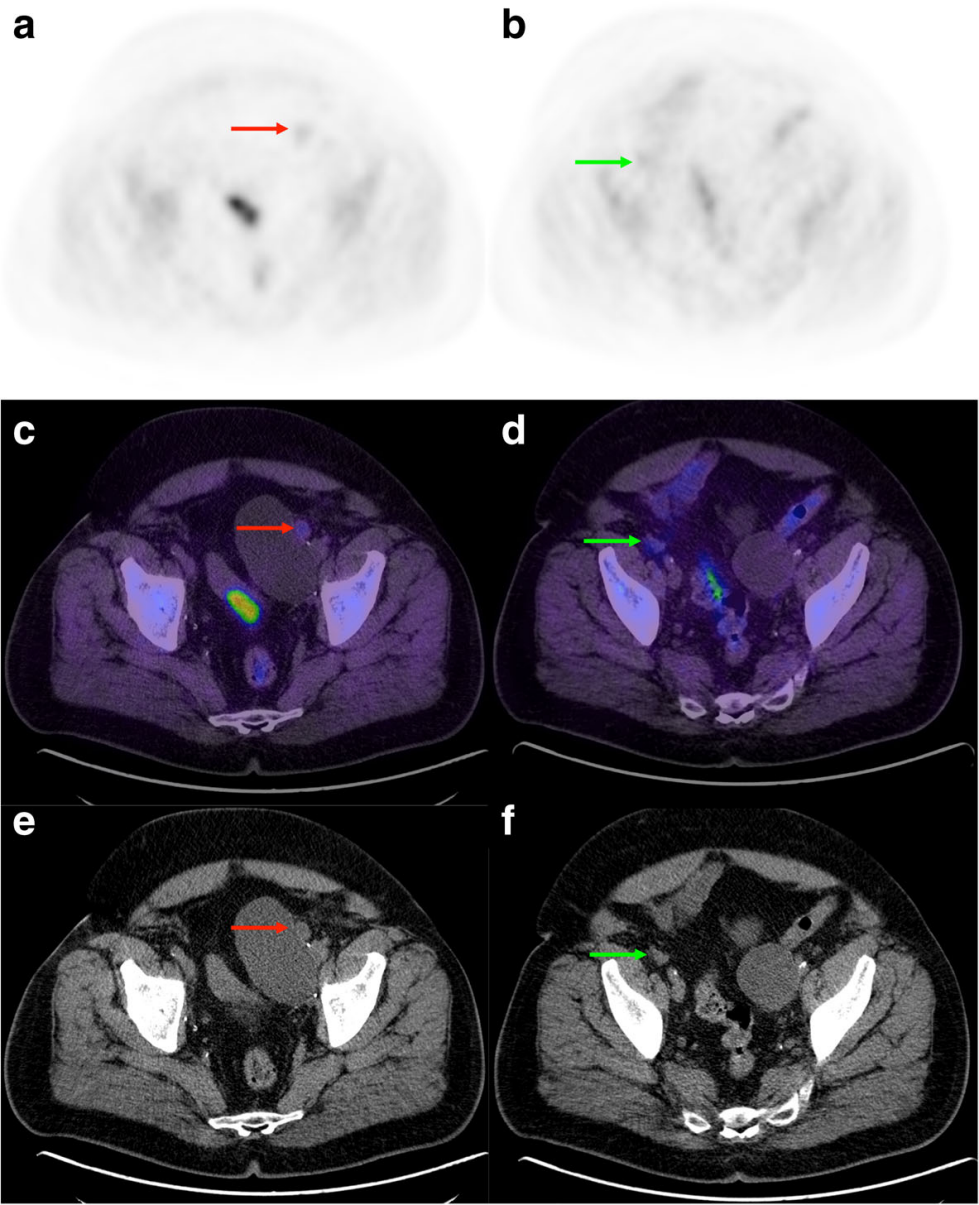
Restaging FCH-PET/CT in a 68 y.o. patient with biochemical relapse following radical prostatectomy. Initially, PCa Gleason sum was 8. PSA was 0.48 mg/L at time of examination. Transaxial PET (**a, b**), fusion (**c, d**) and CT (**e, f**) slices showing faint activity in left paravesical (red arrows) and right external iliac (green arrows) enlarged lymph nodes. However, the very low uptake in the latters was suggestive of a benign process, such as inflammatory or reactive nodes. This study was interpreted as negative and the patient underwent salvage radiotherapy

In the presence of multiple hyperactive lesions, it is the pattern of dissemination thereof, rather than their level of uptake, which is most critical to determine the probability of metastasis. However, in the presence of a solitary lesion in an area consistent with metastatic spread (e.g. external iliac node ipsilateral to the dominant primary lesion), the level of uptake may have more weight in the diagnostic decision-making process. If the uptake of such lesion were only faint, it would be classified as more likely benign. Figure 6 illustrates a case of bilateral pelvic nodes with faint uptake, which were interpreted as likely negative.

Contrary to conventional imaging, there is no size criterion for positivity in PET/CT. The lack of uptake in a node >10 mm makes the latter more likely benign. On the other hand, PET is frequently frankly positive for nodes <10 mm.

### M: Distant compartment assessment

The first organ to assess here is the skeleton, which is by far the commonest site of distant metastasis in PCa. Typically, bone metastases from PCa are sclerotic, but it is not uncommon that lesions are initially lytic, particularly in untreated or high-grade PCa. FCH uptake is variable in bone metastases and has been shown to inversely correlate with bone density [11]. This is consistent with sclerotic lesions being generally less aggressive, of lower tumor cell density, and/or having responded to therapy. Contrary to most other cancers, spread of PCa to the bone can be regional via the venous plexus of Batson, resulting in bone metastases being more frequent in the pelvis and lower spine. In addition to the location, number (approximate when numerous) and uptake features of suspicious lesions, we also comment on their radiological appearance (lytic, sclerotic or mixed), on the presence of metabolic and/or radiological heterogeneity among lesions, as well as on the risk of pathological fracture (with lesions in weight-bearing bones, of lytic appearance, and/or involving more than 2/3 of bone width harboring the highest risk [12]) or other complications such as cord involvement or compression. Additionally, the presence of bony lesions on CT that are not enhancing and are thought to represent benign lesions (such as benign bone islands) or healed metastases should be mentioned, as they could potentially raise suspicion on other radiological exams. Of note, the sagittal view is particularly convenient to assess the spine, as it allows a complete survey thereof over a very few slices. In addition, vertebral fractures are also easily detected in this view.

Other potential sites where metastases needs to be ruled out include the liver, adrenals, lungs and brain, the latter being easier to assess with FCH- than with FDG-PET/CT, as mentioned earlier. The sensitivity of FCH-PET/CT for small liver metastases is reduced, as they either need to exhibit a level of uptake that is superior to that of the liver, or to be large enough so that significant uptake can be evidenced with confidence and despite spill-in from surrounding liver activity. Because liver metastases are relatively infrequent in PCa, this remains a relatively minor drawback of FCH-PET/CT.

If there is no evidence of metastasis, a simple phrase such as *“There are no foci of increased uptake elsewhere that are suspicious for metastases, in particular in the bones”* will concisely summarize relevant negative findings. Because at our institution CT performed as part of a PET/CT procedure is acquired at low amperage, without IV contrast and in free-breathing conditions, our focus is not on trying to find – or exclude the presence of – any small lesion without detectable FCH uptake, which could otherwise be better visualized on a fully diagnostic CT or MRI scan. Considering that the negative predictive value of FCH-PET/CT has been shown to be superior to that of conventional imaging [2, 3], such small lesions without detectable FCH uptake are less likely to be clinically relevant.

### Incidental and accessory findings

An incidental finding is a condition unrelated to that for which the FCH-PET/CT was requested, and which presence was unknown before the procedure. It differs from an accessory finding in that it may potentially alter patient management. FCH uptake is not specific to PCa, and most cancers other that PCa can also exhibit FCH uptake. An FCH-avid lesion in a location that is atypical for PCa spread – particularly in the absence of more typically located metastases elsewhere – should raise the possibility of a synchronous malignancy. Depending on the context of the patient, further investigation of such an incidental finding may or may not be clinically relevant. Other incidental findings that are more easily detected on CT (even on low-dose CT) include potentially life-threatening conditions such as a large vascular aneurysm, a pneumothorax or a cavity effusion.

Accessory findings are abnormalities of lesser clinical interest to the referring physician, which do not fall under the PCa-relevant positive or negative findings, or incidentalomas categories. Virtually any benign lesions, traumatic or inflammatory processes that can demonstrate hypermetabolism with FDG can also exhibit increased FCH uptake to a variable extent. Again and again, it is the distribution pattern of hyperactive lesions that is the most determinant factor in the diagnostic decision-making process. Uptake intensity may modulate the level of suspicion of malignancy, but is not diagnostic by itself. CT appearance is often helpful to increase the level of confidence. For example, an FCH-avid adrenal nodule will confidently be classified as a benign adenoma in the presence of a low attenuation on non-contrast low-dose CT. Other examples of accessory findings include FCH uptake related to osteoarthritis, hilar and mediastinal reactive lymph nodes, reactive bone marrow (diffuse uptake), and traumatic lesions. Accessory findings detected on CT only, i.e. without metabolic correlate, include surgical sequella unrelated to PCa (of which cholecystectomy is probably be the most common), vascular calcifications, steatosis, renal or liver cysts, and old fractures, just to name a few.

A normal structure with an FCH uptake within normal range should not be described. This only lengthens the report, without adding clinical value. Finally, for the reasons mentioned earlier concerning low-dose CT, we do not describe any organ as having a “normal” appearance on CT, as a fully diagnostic CT could contradict this.

### SUV

Tumor SUVs are not systematically reported because they have not consistently been shown to improve diagnostic accuracy over qualitative interpretation. There is no clinically proven FCH SUV cutoff that can rule in or out malignancy. SUV is heavily influenced by the partial volume effect. PET can detect small, even sub-cm metastases, but SUV will inevitably be lower in such lesions. Visual assessment may, at least partly, overcome this limitation (Table 1).

SUVs are useful in research as a continuous quantitative variable to be correlated with tumor or patient characteristics or outcomes in a given population. But, in the clinical setting, the only utility of SUV is to compare uptake values between repeat studies in the same patient, e.g. for therapeutic response assessment. Even then, SUV comparison remains in support of qualitative uptake assessment, and is valid only if the current and prior studies are normalized to a similar SUV upper threshold, implying that the organ of reference’s uptake is stable. While a SUV difference of 30% is generally considered significant, i.e. unlikely due to chance [13], it is our experience that such a difference is easily picked up visually when images are properly normalized. Furthermore, complete molecular response remains a visual criterion, as SUV can virtually never reach zero. Hence, SUV comparisons have a limited added value other than illustrating the amplitude of the lesion’s partial response or progression.

In the end, in the oncologic setting, the foremost important feature of scintigraphic abnormalities, before considering any uptake quantification, is their distribution pattern relative to each other and to the primary tumor. A high-uptake lesion in an uncommon location for a metastasis is most often not one, particularly if no other more typically located metastases are found. Likewise, a definite focus of mild uptake in a rounded sub-cm lymph node in the obturator area ipsilateral to the primary PCa lesion will raise some suspicion of metastasis, regardless of the actual SUV. In both situations, PCa-specific pattern recognition and clinical judgment are key to accurate diagnosis, not SUVs. In our experience, consistent qualitative appreciation of uptake intensity (e.g. as in Table 1) instead of systematically reporting SUVs has many advantages: (1) it avoids pitfalls related to SUV measurements, (2) it minimizes the too often overstated importance of SUVs and (3) it makes the report more concise.

## Report structure

Our reports are divided in 4 sections, as follows:

### Clinical information section

Clinical information available at the time of reporting should be summarized. This includes the relevant information gathered from the requisition form (including the clinical question to be answered), the patient questionnaire and interview, the medical record, or the treating team. Details about the pathology results (including Gleason score), prior and current treatments, other imaging findings, biochemical status (PSA and, if relevant, PSA doubling time) and symptoms that are relevant for the interpretation of the FCH-PET/CT scan should be mentioned.

### Technique section

This section is brief and limited to few sentences. It is not necessary to mention every technical detail that can be found elsewhere, in the standard procedure protocol or patient imaging record, unless there has been a significant protocol deviation that could potentially impact on the quality of the scan. We favor a brief statement about the imaging sequence and technique, the areas surveyed, and the use of contrast or drug if any. For example: *“At the time of FCH administration, we started a 10-min dynamic PET acquisition over the pelvis, which was followed by a whole-body acquisition from vertex to proximal thighs. Both acquisitions were accompanied by a low-dose CT with oral contrast for anatomical correlation and attenuation correction.”*

### Observations section

The observation section describes the relevant positive and negative findings in a TNM sequence, as detailed earlier. For simpler cases, one paragraph is generally sufficient, while for more complex cases T, N and M compartments can be separated in distinct paragraphs. The emphasis is on the description of the findings, rather than opinions.

If there were any incidental finding, its description would come thereafter, in a separate paragraph. This is followed by description of accessory findings, starting with “*Accessorily, …*”. Because accessory findings are most of the time benign and unrelated to the oncological condition, their description should be succinct. Further, for these we tend to formulate a diagnostic opinion together with the description within the Observations section, particularly for obviously benign findings that would be superfluous to point out again in the conclusion. An example of this could be: “*There is mild uptake in multiple hilar and mediastinal lymph nodes, which are symmetric in distribution and thus likely of inflammatory or granulomatous etiology.*”

In the case of a repeat study, to assess either the evolution under observation or the therapeutic response, the description should focus on the metabolic (and secondarily anatomic) evolution of previously seen PCa lesions, as well as the apparition of new suspicious lesions if any. We would also comment on the homogeneity or heterogeneity of lesions’ evolution or response. Here there is no need to mention again all accessory findings if they are stable and clinically irrelevant. A simple statement such as “*The remainder of the scan shows the same accessory findings as previously described. No other significant changes are seen as compared to the previous study*” can be used instead.

### Conclusion section

This is the most important section of the report, and in the majority of cases, the only one the referring physician will ever read. Conclusion should straightforwardly answer the clinical question, which is typically to stage or restage PCa. Hence, the first sentence(s) should formulate as concisely as possible an opinion on what the TNM stage is. Completely equivocal opinions should be avoided as much as possible. In those more difficult cases, the arguments for and against a given hypothesis can be summarized in the conclusion, ending up with a likelihood statement about the hypothesis. These arguments may include clinical information affecting the probability of a positive (or negative) finding being truly positive (or negative). For example, it has been demonstrated that a high Gleason score, a high PSA and a short PSA doubling time are associated with higher sensitivity of FCH-PET/CT [4]. Hence, an equivocal finding based on FCH-PET/CT images alone, such as a solitary pelvic lymph with a rather mild FCH uptake, could be interpreted as more likely to represent metastasis in a patient with any of these characteristics, or less likely to in a patient with none.

Opinion on the probable nature of any incidental finding is given thereafter. However, accessory findings are only mentioned in the conclusion if they are likely to alter patient care. For example, we seldom mention the presence of cholecystectomy sequela in the Conclusion section of an oncological PET/CT report.

Organization of conclusion in a bulleted list is preferred and improves readability. Repeating findings’ description is avoided as much as possible. Differential diagnoses are typically limited to the few most likely ones, in order of probability. When relevant, appropriate next diagnostic steps are proposed. For example, a typical conclusion looks like this:

- *The findings are consistent with a primary prostate cancer lesion extending over the middle and apical sextants of the left prostatic lobe, accompanied by two nodal metastases in the left external iliac area.*
- *No evidence of osseous or visceral metastases.*
- *Incidental finding of a thyroid nodule with moderate uptake in the right thyroid lobe, for which a primary thyroid malignancy cannot be ruled out. This could further be investigated with ultrasound and fine-needle biopsy if clinically relevant in the setting of this patient.*

If FCH-PET/CT is performed for therapeutic response assessment, the conclusion could be as simple as:

- *The findings are consistent with a complete metabolic response.*

## Conclusion

In summary, FCH-PET/CT for PCa is read and reported in a similar fashion as oncological FDG-PET/CT. For the experienced FDG-PET/CT reader, the learning curve is rapid, and involves mastering the normal biodistribution of FCH, the dissemination pattern of PCa, and benign pathologies exhibiting FCH uptake (largely overlapping with those associated with FDG uptake). Reviewing the dynamic PET images may be challenging at first, as not all software viewers are user-friendly for this purpose. However, there are overall more similarities than differences between reporting oncological FCH- and FDG-PET/CT. For both, we encourage adopting a problem-oriented approach based on the TNM system, in combination with a concise reporting style clearly addressing the clinical question upfront, both in the Observations and Conclusion sections. In our opinion, this makes PET/CT reading more efficient for the nuclear medicine physician and radiologist, and report clearer and more clinically relevant for the referring physician or surgeon.
